# Childhood sun safety at different ages: relations between parental sun protection behavior towards their child and children’s own sun protection behavior

**DOI:** 10.1186/s12889-019-7382-0

**Published:** 2019-08-05

**Authors:** Karlijn Thoonen, Francine Schneider, Math Candel, Hein de Vries, Liesbeth van Osch

**Affiliations:** 1 Department of Health Promotion, School CAPHRI, Maastricht University, Maastricht, the Netherlands; 2 Department of Methodology and Statistics School CAPHRI, Maastricht University, Maastricht, the Netherlands

**Keywords:** Skin neoplasms, Primary prevention, Health behavior, Children, Parents

## Abstract

**Background:**

Sunburns during childhood are strongly associated with development of melanoma in later life. While parents play an important role in children’s sun protection, insight in possible shifts in behavioral responsibility from parents towards their children and the possible effect of children’s sex is important for targeting sun safety interventions throughout childhood and adolescence.

**Methods:**

This cross-sectional survey study was conducted among a representative sample of Dutch parents (*N* = 1053) of children aged between 4 and 13 years old. Questionnaires measured both parental and children’s own sun safety behavior during planned (e.g. going to the beach) and incidental (e.g. bycicling) sun exposure situations. Analyses of variance were used to test for age group differences and linear regression models were computed to detect behavioral shifts in executive behavior.

**Results:**

Parents applied all sun safety behaviors (i.e. sunscreen use, wearing UV-protective clothing and seeking shade) more often on younger children, except for supportive behavior (facilitating children’s own sun safety behavior), which remained relatively stable over the years. Older children and girls were more likely to execute sun safety behaviors themselves. A behavioral shift was found in wearing UV-protective clothing during planned situations among 11 year old children. For other behaviors, shifts were predicted after the age of 13.

**Conclusions:**

Older children execute sun safety behaviors more often than younger children, although they still largely depend on their parents’ protection. Specific attention for boys in the primary school years, and for both boys and girls in the years adjacent to adolescence is warranted in skin cancer prevention interventions.

**Electronic supplementary material:**

The online version of this article (10.1186/s12889-019-7382-0) contains supplementary material, which is available to authorized users.

## Background

The incidence of both melanoma and nonmelanoma skin cancer types among fair-skinned populations is increasing rapidly worldwide [1–4]. Factors that have been associated with these increased incidence rates are all related to Ultraviolet Radiation (UVR) exposure. Increased sun exposure behaviors during leisure-time activities, the preference for wearing less covered clothing and increased exposure to tanning beds, influenced by changed attitudes in which people are more in favor of getting a tan and enjoying the sun are important factors related to increasing skin cancer types [3, 5]. Moreover, sun exposure and sunburns during the first 10 to 15 years of life have proven to play an important role in the etiology of all skin cancer types [6, 7] and especially melanoma [8–11] since children’s skin is more sensitive to UVR. Even though sun exposure seems to be distributed equally over a person’s lifetime, prevention of excessive sun exposure and sunburns during childhood is ought to start during early childhood [12]. Children are largely dependent of their parental sun protection, which makes them a particular vulnerable group [13]. Moreover, childhood is an important phase in which health behaviors such as sun protection should be established [14], to increase the likelihood of habitual behavior in later life [15]. To prevent children from getting sunburnt, various precautions can be taken, in which parents play an important role. Application of sunscreen, wearing UV-protective clothing (including a hat and sunglasses) and seeking shade are the most important and recommended sun safe behaviors [5, 16, 17].

Although clear guidelines exist with regard to sun protection of children, the prevalence of sunburns is still high, with studies reporting 28 to 52% of children suffering one or multiple sunburns during the past 12 months [16, 18–20]. Although children in general are at high risk of getting sunburnt, several subgroups need specific attention. Boys in general seem to be more exposed to UVR [21], use less sun safe measures [22, 23] and have more reported sunburns during childhood [24, 25] than girls. Moreover, older children, primarily from the age of 8, are often less protected against UVR than younger children, and more sunburns are reported [16, 17, 23, 26–28]. Possible explanations for the finding that children are less protected as they get older is that older children spend more unsupervised time outside the house and gain self-responsibility and independence, which makes parental influence on children’s behavior less probable [15, 16, 29, 30]. Furthermore, positive sun-protective attitudes and behaviors of children seem to weaken when children reach adolescence [28]. However, parental sun safe attitudes, beliefs and behavior are considered of great influence, even when a child grows older [30–32]. For example, Behrens and colleagues [33] found that parental attitudes favoring a tanned skin accounted for an 85% increase in sunburn risk of children aged 13 and above, while this effect was not seen among children aged between 0 and 6 years old. Even though parental influences seemingly play a vital role in sun safety behavior, specific insight in the occurrence of sun safety behavior of children themselves as they grow older are lacking. Although alterations in children’s behavioral responsibility seem to occur within other health behaviors such as medicine use, healthy food intake or tooth brushing [34–37], occurrence of possible behavioral shifts concerning sun safety behavior have not yet been investigated.

The primary school age is an important period in which children start to adopt and develop self-responsibility and autonomy over their health behavior, a process that is continued throughout adolescence and is thought to result in the formation of habitual behavior [38, 39]. During this phase children learn that the environment expects them to start controlling their own behavior and that their individual freedom expands [29, 40]. Given the fact that sunburn incidence rates are high among older children, gaining insight in possible behavioral shifts from parental executive to children’s own executive behavior is imperative for targeting interventions towards specific age groups and developing tailored content. Furthermore, based on existing differences in UVR exposure and sunburn incidence between boys and girls, examining the role of gender in the occurrence of sun safe behaviors of both parents and children as the child grows older is of great importance and can contribute to developing tailored sun safety interventions. This study therefore aims to gain insight in executive sun safe behaviors of parents and their child and investigates: 1. the relations between children’s age and sex and the occurrence of extensive sun safe behaviors of both parents towards their child and children themselves in the age range of 4 to 13 years; and 2. the relations between children’s age and sex on the one hand and the differences in execution of these behaviors between parents and their child on the other hand (i.e. investigation of when and how a possible behavioral shift takes place).

## Methods

### Participants and procedures

A cross-sectional survey study was conducted among Dutch parents (*n* = 1053) of children aged between 4 to 13 years, in November 2016. The Dutch research organization TNS-KANTAR (http://www.tns-nipo.com/) invited a sample of the Dutch population (*n* = 1222), representative with regard to education and income of the parents and age of the child, to participate via e-mail. Parents were eligible for participation if they had one or more children within the age range of the study (4–12 years). Participants were invited to fill in an online questionnaire about sun safety behaviors regarding their youngest child. Respondents received one reminder e-mail. In total, 1053 parents filled in the questionnaire (86%). TNS-KANTAR works with a permanent panel of respondents, who receive small incentives in terms of vouchers for their participation in studies. The data for this study were collected in November 2016. This study was exempt from review from the medical ethical commission, since respondents were not subjected to procedures, activities or behavioral requests [41]. Respondents were not part of a vulnerable group. TNS-KANTAR retrieved online informed consent of all respondents beforehand. Since respondents were part of a survey panel, informed consent had to be given by definition [42]. In accordance with the European Union-wide law on data protection (General Data Protection Regulation), the data in this study was not identifiable nor translatable to the respondents [43].

### Behavioral measures

The online questionnaire contained demographic questions concerning, among others, sex and age of the child. Additionally, the frequency of execution of three sun safe behaviors during the previous summer season was asked about, for both the parent towards the child and the child him/herself. Sun safe behaviors consisted of sunscreen use, wearing UV-protective clothing, and seeking shade. Behavioral measures were based on Dutch guidelines regarding sun safety [44] and a previously validated questionnaire [45]. Questions about children’s executive behavior were based on parental perceptions regarding their child’s performance. Furthermore, supportive behavior of parents was also asked for, which consisted of advising their child about sun safety, facilitating sun safety measures and checking whether the child applied sun safety behaviors. Explanatory text for all sun safe behaviors was used according to guidelines from the Dutch Cancer Society. A full overview of the outcome variables is provided in Table 1.

**Table 1:** Sun safe behaviors of both parents and the child himself or herself

	Behaviors	N of items	Exemplary item
Executive behaviors: 1. Sunscreen use	*Parents towards their child* Primary behavior - Sunscreen application in general Sub-behaviors - Using a minimum of SPF 30 - Applying sunscreen at least 30 min before sun exposure - Reapplying sunscreen every two hours	(8 items; 4 in planned situations, 4 in incidental situations)	*‘To what extent did you make sure your child was sufficiently protected with sunscreen when he/she was at the beach or swimming pool/engaged in other outdoor activities?’* *(1. Never - 5. Always)*
	*Parental perception of child’s behavior* - Applying sufficient sunscreen	(2 items; 1 in planned situations, 1 in incidental situations)	*‘My child applies sunscreen sufficiently when he/she goes to the beach or swimming pool/engaged in other outdoor activities.’* *(1. Never - 5. Always, 6. I don’t know)*
2. Wearing UV-protective clothing	*Parents towards their child* Primary behavior - Wearing UV-protective clothing Sub-behaviors - Wearing a long-sleeved t-shirt - Wearing a cap or hat - Wearing sunglasses	(8 items; 4 in planned situations, 4 in incidental situations)	*‘To what extent did you make sure your child was wearing UV-protective clothing when he/she was at the beach or swimming pool/engaged in other outdoor activities?’* *(1. Never - 5. Always)*
	*Parental perception of child’s behavior* - Wearing UV protective clothing	(2 items; 1 in planned situations, 1 in incidental situations)	*‘My child puts on UV-protective clothing when he/she goes to the beach or swimming pool/engaged in other outdoor activities.’* *(1. Never - 5. Always, 6. I don’t know)*
3. Seeking shade	*Parents towards their child* - Staying in the shade between 12 and 3 PM	(2 items; 1 in planned situations, 1 in incidental situations)	*‘To what extent did you make sure your child was in the shade between 12 and 3 PM when he/she was at the beach or swimming pool/engaged in other outdoor activities?’* *(1. Never - 5. Always)*
	*Parental perception of child’s behavior* - Seeking shade during a sunny day	(2 items; 1 in planned situations, 1 in incidental situations)	*‘My child seeks shade when he/she goes to the beach or swimming pool/engaged in other outdoor activities.’* *(1. Never - 5. Always, 6. I don’t know)*
Supportive behavior	*Parents towards their child* - Supporting the child’s own executive behavior	(2 items; 1 in planned situations, 1 in incidental situations)	*‘To what extent did you support your child to make sure he/she could protect himself or herself sufficiently?’* *(1. Never - 5. Always)*

### Sun exposure situations

Both the three executive sun safety behaviors of both parents and their child, and supportive behavior of the parents, were assessed in planned (e.g. going to the beach or the swimming pool) and incidental (e.g. being outside for other recreational purposes such as playing, cycling or walking) sun exposure situations (see Table 1 and the questionnaire in the Additional file 1).

### Statistical analyses

Descriptive statistics were calculated for sex (%) and age (M; SD) of the child and performance of sun safety behaviors of both parents and their child (M; SD). The sub-behavior items within parental sunscreen use and putting on UV-protective clothing were not used in the analyses considering the fact that these behaviors are distinctive and therefore were not suitable for grouping. Missing values were coded when parents were unaware of the executive behavior of their child. For the first research question, age of the child was categorized according to the Dutch primary school system. The youngest age group consisted of children between 4 and 6 years old (elementary school, grade 1 to 3), intermediate-aged children were between 7 and 9 years old (middle school, grade 4 to 6) and the oldest children were aged between 10 and 13 years old (senior school, grade 7 to 8). For the first research question, two-way analyses of variance (ANOVA) were first performed to test for effects of interaction between age and sex of the child on the sun safety behaviors. When the null hypothesis of no interaction was rejected (*p* < .05), one-way analyses of variance (ANOVA) were performed for boys and girls separately. Post hoc Tukey HSD tests were then used to test for differences between age groups. When the null hypothesis of no interaction was accepted, two-way analyses of variance were again performed after eliminating the interaction term. Sidak post hoc comparisons where then used for comparisons of the age groups (*p* < .05). Possible sex differences in sun safety behaviors were then also investigated using the two-way ANOVA without the interaction term (*p* < .05). To examine the second research question relating to the development of the difference in sun safety behaviors of parents towards their child and children themselves, linear regression analyses were conducted. Difference scores involving mean scores of sunscreen use, wearing UV-protective clothing and seeking shade of the parents minus the mean scores of the child were calculated to test whether this difference between parental and child’s behavior decreases linearly as the child’s age increases. The difference scores were computed for both planned and incidental sun exposure situations. To test for linearity, nine dummy variables for age, with the youngest age of 4 years as reference category, were formed for all remaining ages. Linear age models were then compared with saturated age models in which the nine dummy variables for age were put into the model. Linear age models were accepted when a significance value of *p* > .05 was reached. By extrapolating the regression results, possible behavioral shifts at later ages were predicted. To test for possible sex differences in the development of difference scores across age of the child, linear regression analyses were again performed, but then with the interaction between age and sex as additional predictor. Statistical analyses were performed using IBM SPSS Statistics for Windows, Version 21.0 (IBM Corp., 2013).

## Results

### Sample characteristics and executive behaviors of parents and their child

All parents (*N* = 1053) answered the questions, for 542 boys (51.5%) and 510 girls (48.5%). The children’s mean age was 7.88 years (SD = 2.59). Of the three sun protection behaviors, sunscreen use was the most preferred method in both planned and incidental sun exposure situations by both parents and their child. Among the children, almost all behaviors were more frequently executed by girls than boys. Furthermore, supportive behavior was also frequently applied by parents in both situations (see Table 2).

**Table 2:** Frequencies of overall sun safe behaviors and sun safe behavior differences with regards to sex and age using two-way and one-way ANOVA

		Parents								Children							
		Overall (*N* = 1053) (M;SD)		Youngest (4–6)		Intermediate (7–9)		Oldest (10–13)		Overall (M;SD)		Youngest (4–6)		Intermediate (7–9)		Oldest (10–13)	
		Boys	Girls	Boys	Girls	Boys	Girls	Boys	Girls	Boys	Girls	Boys	Girls	Boys	Girls	Boys	Girls
Applying sunscreen	Planned	4.36 (.84)	4.41 (.76)	4.54^a^	4.42	4.36^a^	4.43	4.12^b^	4.35	2.51 (1.42)	3.08 (1.43)	2.20^a^		2.80^b^		3.53^c^	
	Incidental	3.80 (.96)	3.95 (.88)	4.07^a^	4.06^D^	3.74^b^	3.96^DE^	3.48^c^	3.82^E^	2.25 (1.27)	2.70 (1.30)	2.07^a^		2.44^b^		3.03^c^	
Wearing UV-protective clothing	Planned	3.33 (.90)	3.27 (.96)	3.50^a^		3.25^b^		3.11^b^		2.60 (1.22)	2.85 (1.20)	2.37^a^		2.77^b^		3.09^c^	
	Incidental	3.42 (.99)	3.30 (.99)	3.53^a^		3.33^b^		3.20^b^		2.48 (1.14)	2.74 (1.14)	2.41^a^		2.59^a^		2.87^c^	
Seeking shade	Planned	3.21 (1.00)	3.26 (.94)	3.42^a^		3.13^b^		3.12^b^		2.03 (1.19)	2.19 (1.21)	1.98^a^		2.09^a^		2.31^b^	
	Incidental	3.12 (1.02)	3.13 (.98)	3.34^a^		3.04^b^		2.95^b^		2.19 (1.22)	2.28 (2.20)	2.03	2.16^D^	2.27	2.12^D^	2.31	2.59^E^
Supportive behavior	Planned	4.31 (1.11)	4.34 (.95)	4.39		4.26		4.36									
	Incidental	4.02 (1.14)	4.06 (1.00)	4.23^a^		3.86^b^		4.01^b^									

Levels with different superscripts are significantly different in Tukey’s HSD test when one-way ANOVA was performed and in Sidak’s test when two-way ANOVA was performed (*p* < 0.05). Analyses were done separately for planned and incidental situations, and, in the case of an interaction between sex and age, separately for boys (using normal letters as superscript) and girls (using capital letters as superscript)

### Relations between child’s age, sex and sunscreen use

The relation of age and sex of the child on the one hand and parental sunscreen use on the other was examined for both planned and incidental sun exposure situations. An interaction between age and sex was found for both planned (F (2, 1095) = 4.309, *p* = .014) and incidental (F (2, 1095) = 3.516, *p* = .030) situations. Parents applied sunscreen more frequently on youngest and intermediate-aged boys compared to older boys in both situations. Subsequently, parents applied sunscreen more often on younger aged girls than older ones, but only during incidental situations (Table 2).

There was no interaction between age and sex as regards the child’s own sunscreen use. Differences between the three age groups however occurred in both planned (F (2, 1087) = 89.300, *p* < .001) and incidental (F (2, 1091) = 55.621, *p* < .001) situations. In both sun exposure situations, older children applied sunscreen more often than the youngest and intermediate-aged children, and intermediate-aged children performed sunscreen use more often than the youngest children. A full overview of results is provided in Table 2. Moreover, girls more frequently applied sunscreen than boys in both planned (F (1, 1087) = 36.218, *p* < .001) and incidental (F (1, 1091) = 27.351, *p* < .001) situations.

### Relations between child’s age, sex and difference in child’s and parental sunscreen use

**Fig. 1:**
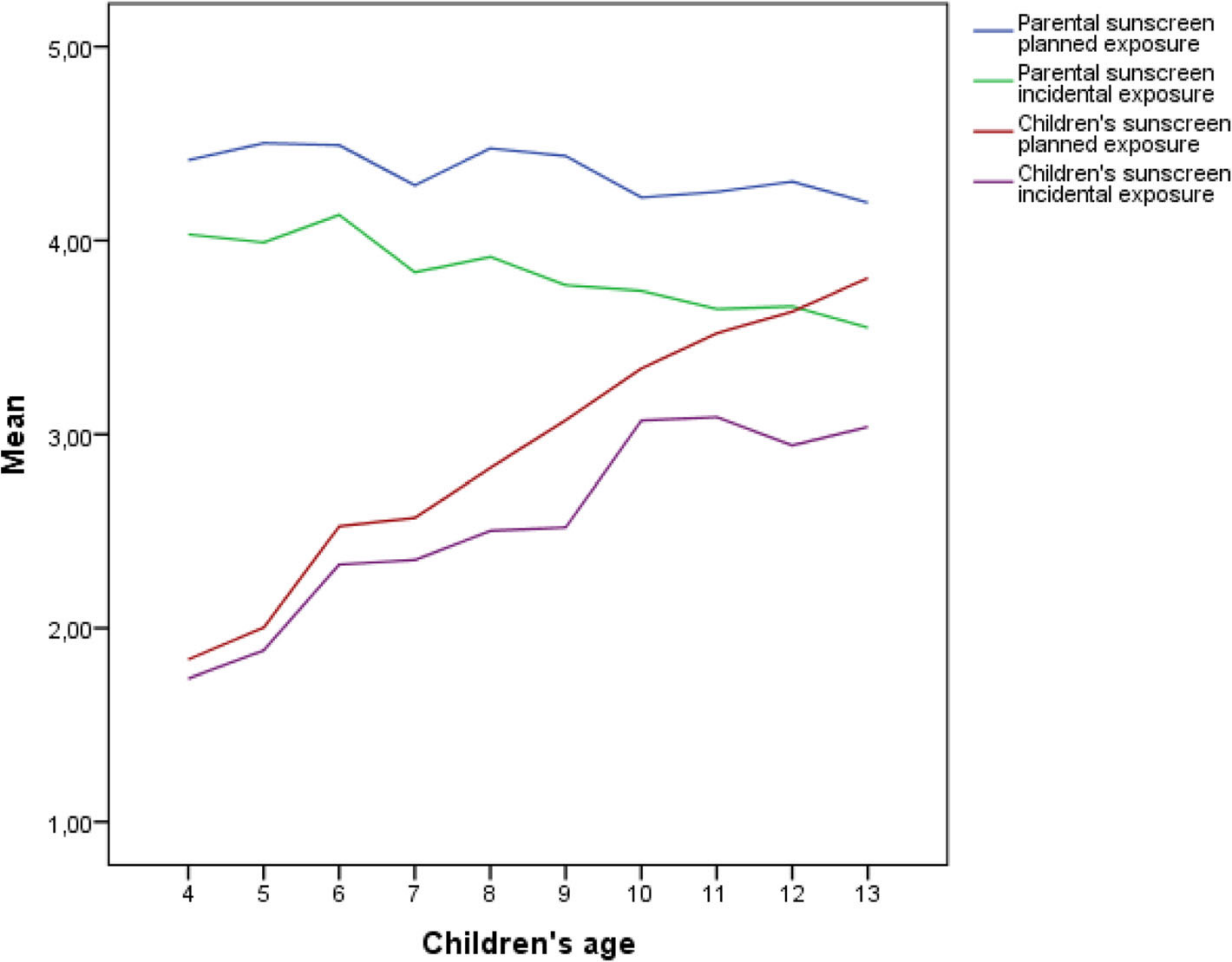
Sunscreen use of parents towards their children and sunscreen use of the child himself or herself in planned and incidental sun exposure situations

After performing regression analyses, the assumption of linear age effects was accepted for the relationship between the difference in sunscreen use of parents and their child with the age of the child, in both planned (*β =* −.26, *t*(− 14.25), *p* < .001) and incidental (*β =* −.22, *t*(− 13.46), *p* < .001) situations. This analysis shows that difference in sunscreen use between parents and their child decreases for older children, which might be caused by decreased application of sunscreen by parents and increased sunscreen use by children themselves as they grow older (see Fig. 1 depicting the trend across the child’s age of both parental and children’s sunscreen use at planned and incidental sun exposure situations). Across the age range studied, no shifts in sunscreen use, indicated by the child taking primary responsibility for sunscreen use (i.e. by applying sunscreen more often than the parents), were observed in either planned or incidental situations. However, based on extrapolation of the regression results, a shift in sunscreen use can be predicted approximately at the age of 14 years.

### Relations between child’s age, sex and clothing behavior

No interaction of age and sex on the application of UV-protective clothing of parents towards their child was found. After eliminating the interaction term, however, age group differences were found in both planned (F (2, 1097) = 15.195, *p* < .001) and incidental (F (2, 1097) = 10.112, *p* < .001) situations. Parents put on UV-protective clothing more often on the youngest compared to intermediate-aged and oldest children in both situations (Table 2). Parents did not differ in putting on UV-protective clothing for boys and girls.

Again, no interaction between age and sex as regards child’s own clothing behavior was found. However, age group differences were also found in both planned (F (2, 1067) = 46.394, *p* < .001) and incidental (F (2, 1082) = 15.811, *p* < .001) situations. In planned situations, older children wore UV-protective clothes more often than all younger aged children, and intermediate-aged children executed the behavior more often than the youngest children. In incidental situations, older children performed clothing behavior most often compared to all younger children. See Table 2 for more detailed results. Moreover, girls put on protective clothing more often than boys in both planned (F (1, 1067) = 9.380, *p* = .002) and incidental (F (1, 1082) = 12.137, *p* = .001) situations.

### Relations between child’s age, sex and difference in child’s and parental clothing behavior

**Fig. 2:**
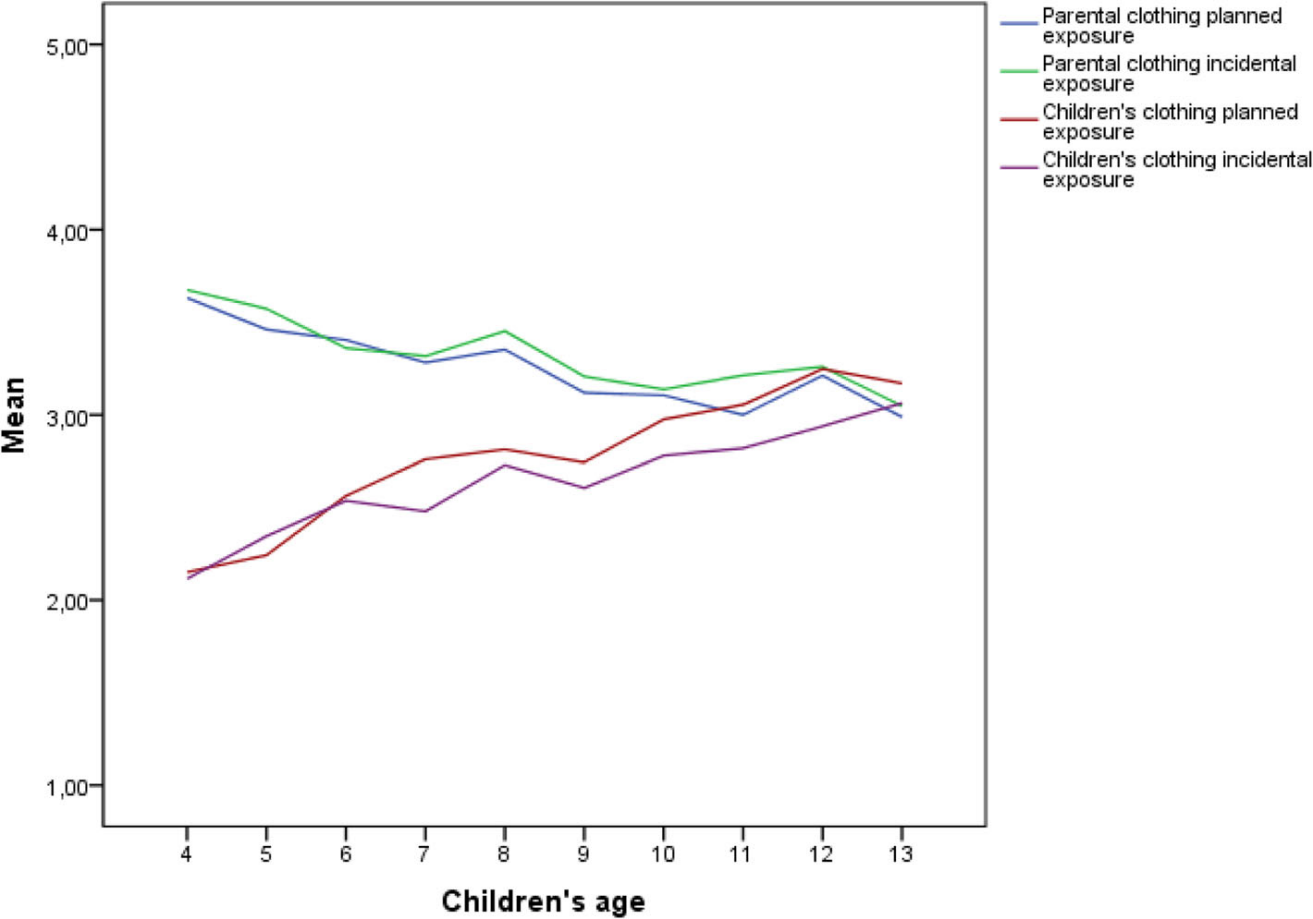
Putting on UV-protective clothing of parents to their child and wearing UV-protective clothing of the child himself or herself in planned and incidental sun exposure situations

The assumption of linear age effects was also accepted for the relationship between differences in wearing UV-protective clothing of parents and their child with the age of the child (see Fig. 2). In planned situations (*β =* −.18, *t*(− 12.18), *p =* .000), we detected a behavioral shift at the age of 11, after which the child executes clothing behavior more often than the parents. In incidental situations (*β =* −.14, *t*(− 9.46), *p* < .001), a shift is predicted by extrapolating the regression results, approximately at the age of 14 years.

### Relations between child’s age, sex and seeking shade

No interaction effects between age and sex were found for parental shade seeking. Age group differences concerning seeking shade were found in both planned (F (2, 1097) = 11.284, *p* < .001) and incidental (F (2, 1097) = 15.907, *p* < .001) situations; parents keep their younger children more often in the shade than intermediate aged and oldest children. There were no differences observed in seeking shade for girls or boys.

For children themselves, an interaction between age and sex was found only for seeking shade in incidental situations (F (2, 1087) = 3.012, *p* = .05). Older girls sought shade more often than younger and intermediate-aged girls. Furthermore, age group differences were found in planned situations (F (2, 1083) = 7.211, *p* = .001), in which older children sought shade more often than all younger aged children (see Table 2 for details).

### Relations between child’s age, sex and difference in child’s and parental shade-seeking behavior

**Fig. 3:**
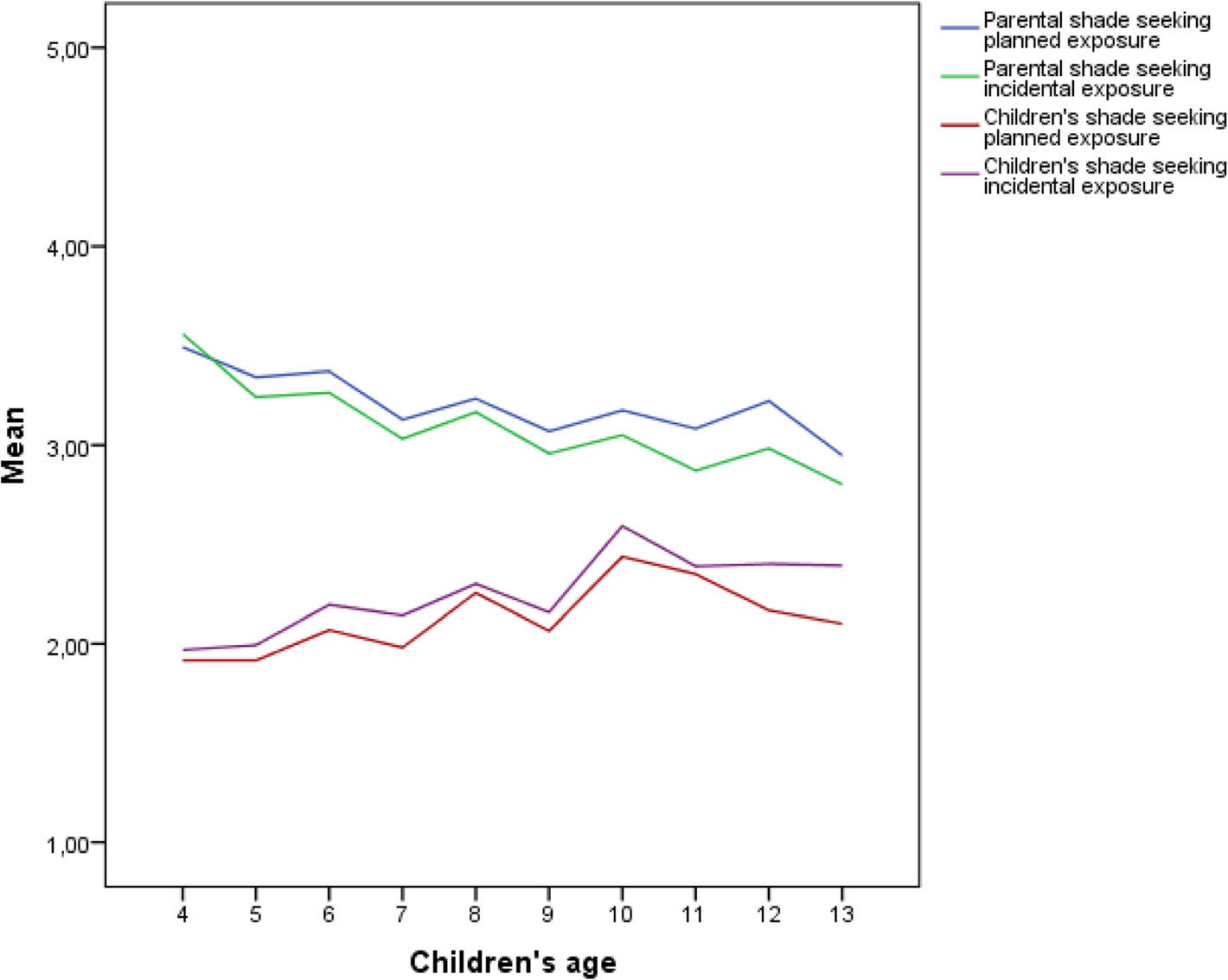
Parents seeking shade for their child and shade-seeking behavior of the child himself or herself in planned and incidental sun exposure situations

There was again a linear relation of age with the difference in shade-seeking behavior in both planned (*β =* −.09, *t*(− 5.5), *p* < .001) and incidental (*β =* −.12, *t*(− 8.0), *p* < .001) situations (see Fig. 3), suggesting that the difference in shade-seeking behavior between parents and their child might diminish as the child grows older. However, before the age of 13, children did not appear to seek shade more often than their parents. No shift in this behavior is predicted until the age of 14, meaning that the difference in shade-seeking behavior of parents and children decreases slowly.

### Sex and the relation of age with differences in sun safety behaviors between parents and their child

For all sun safety behaviors, no significant differences between boys and girls concerning the relation between age and the difference in sun safety behaviors between parents and their child were observed. This indicates that children’s sex does not play a role in the prediction of the difference between execution of sun safe behavior of parents and their child as the child grows older.

### Supportive behavior of parents

Finally, no interaction between age and sex was present for supportive behavior of parents in both situations. Differences were observed between the three age groups (F (2, 1097) = 10.553, *p* < .001) only in incidental sun exposure situations, which showed that parents more frequently perform supportive behavior among the youngest compared to all older children. See Table 2 for detailed results. Furthermore, parents did not apply supportive behavior more often among boys or girls.

## Discussion

This study examined the differences between parents and their child concerning the execution of sun safe behaviors in the context of an increasing age of the child. Furthermore, effects of sex and age of the child on the development of these behaviors were investigated. When comparing sun safety behaviors, for both parents and children sunscreen use appeared the most frequently applied, followed by wearing UV-protective clothing and seeking shade, which corresponds with previous studies [46–48]. In addition, parents with young children applied sun safety measures significantly more often than parents with older children, which also corresponds with earlier work [17, 23]. Additionally, older children were more likely to execute sun safe behaviors themselves. Interestingly, these results contrast with previous work, which state that sun protection of children themselves declines as children grow older [13, 23, 28]. Weakened parental encouragement towards the children as they grew older was mentioned as an important cause for this decline. In our study, supportive behavior of parents remained relatively stable throughout the age range studied, which could have stimulated older children’s sense of their own behavioral responsibility.

Furthermore, a possible effect of children’s sex on sun safety behaviors was examined. Parents seem to apply sunscreen less frequently on older boys than younger ones in both planned and incidental situations, while age group differences for girls were only observed in incidental situations. Moreover, girls themselves executed all sun safety behaviors more often than boys in both situations. For shade-seeking behavior, older girls more often stayed in the shade than younger girls, which is interesting since literature concerning sun safety behavior among adolescents reveals that especially girls gain a desire to tan and are more likely to use tanning beds than boys, occurring around the age of 13 to 16 [19, 46, 49]. Based on these results, stimulating sun safety behavior for boys during early childhood deserves specific attention.

The overall results concerning the development of parental and child’s sun safe behavior indicate that during the age of 4 to 13 years, children increasingly apply sun safety behaviors, but largely depend on their parents’ protection. The fact that children’s sun safety is strongly related to adequate parental sun safety practices during early childhood is in line with previous studies [17, 26]. Additionally, we found a shift in which children put on UV-protective clothing more often than parents do for them before the age of 13 in planned sun exposure situations. Shifts in other sun safety behaviors were subsequently mostly predicted after the age of 13, when children transition into adolescence [50]. Intervening on enhancing sun safety behaviors during this stage and some time before may be imperative for establishing sun safety behaviors in later life. This is accentuated by the fact that, at the onset of adolescence, increased levels of self-consciousness and internalization of norms and values develop [51], children start to form their own personal identity, and start to differentiate from their families [52]. Moreover, before children reach adolescence, they are still prone to adopting their parents’ values, which makes this specific age even more important to take into account [33]. Nonetheless, children in the age of 4 to 12 years frequently get sunburnt, spend more time outdoors than adolescents and, from the age of 8, gain understanding in the influence they have on their own behavior [14, 29], which makes them an important target group as well. Additionally, from a behavioral development perspective, it is important that children learn how to execute sun protective measures at an early age, rather than get familiar with these behaviors in later life, since unhealthy habits then may already have been established [15, 53]. During the primary school years, stimulating an internal locus of control concerning health behaviors can enhance self-responsibility [54, 55]. Sun safety interventions should therefore target both parents and children during the primary school age with a specific focus on boys. Parental influence is significant and life-long habits start to form, and children start gaining insight in their own health behaviors and can therefore be made aware about importance of sun safety. Additionally, when children transition into adolescence, interventions are of equal relevance, with a strong emphasis on self-responsibility.

There are a few limitations to this study that should be mentioned. First, children’s own sun safety behavior in this study consisted of parental perception of their execution, which may lower the validity of the outcome measure, even though parental perceptions of executive behaviors of their children is the most commonly used method in measuring sun safety behavior [28]. Moreover, frequency of sun safe behaviors was measured using retrospective self-report questions, which may limit the accuracy of behavioral outcomes because of social desirability and the possibility of recall bias [31, 56]. To enhance objectification of sun safety behaviors, future studies should include personal dosimetry measures, preferably together with behavioral diaries [57]. Moreover, combining objective measures with self-reported data allows for detecting risk situations regarding UVR exposure. Nevertheless, self-report measures remain a commonly used method for sun safety behaviors, with correlations among actual sun safe behaviors ranging from low to moderately positive [57, 58]. A further limitation is that children’s age in this study was limited to 13 years, while important behavioral shifts seemed to occur after this age. Even though statistical analyses allowed for prediction of these shifts, extrapolation based on cross-sectional data is less valid compared to time-series data. For future research, a within-subjects design using longitudinal data is essential to investigate behavioral changes of both parents and their child over time. Since findings about sun protection behavior are weather dependent [45] and questions were asked regarding the past summer season, future studies are necessary to allow for seasonality. Additionally, elaborate data about objective UVR exposure of both parents and children in the Netherlands over time, is needed to target childhood sun safety interventions more accurately. Moreover, since children can be influenced by their caretaker’s health behaviors and tend to imitate what they see [13, 27, 59], future studies might also include questions regarding parental modelling and its effects on children’s own executive behavior.

The findings in this study concerning sun safe behaviors of children suggest that parental behavior declines whereas children’s own executive behavior increases as a child grows older. Moreover, girls seem to protect themselves better than boys and parents apply sunscreen less on older boys than younger ones. However, children are not yet taking main responsibility for their sun safe behavior during the assessed time interval (4–13 years), given the fact that a behavioral shift was only apparent in wearing UV-protective clothing during planned situations. These results lead to the recommendation that sun safety interventions during the primary school years should be focusing on both parents and their children, in which specific stimulation of sun protection among boys is warranted. In addition, since we predicted behavioral shifts after the age of 13, the age from which children reach adolescence suggests that this is an important target group as well for emphasizing that children take self-responsibility by providing them with advice and suggestions. However, follow-up data is imperative to consolidate the findings from this study and examine possible short-term developments.

## Conclusions

In conclusion, the results from this study emphasizes the importance of targeting sun safety interventions on both parents and their children during the primary school phase. Moreover, this study confirms that primary school-aged boys are an important target group for sun safety interventions as they are less protected than girls, while also highlighting the relevance of targeting interventions around the pre-adolescence phase for both boys and girls. Follow-up data on sun protection behavior is however recommended to allow for weather dependent behavior and to confirm these findings.

## Additional file

Additional file 1: Questionnaire. All questions asked in the questionnaire, in English. (DOCX 15 kb)

## Data Availability

The datasets used and/or analysed during the current study are available from the corresponding author on reasonable request.

## Abbreviations

ANOVA: Analyses of Variance
UVR: Ultraviolet Radiation
