# Supplementary material for: Childhood sun safety at different ages: relations between parental sun protection behavior towards their child and children’s own sun protection behavior
Source: BMC Public Health. 2019 Aug 5;19:1044. doi: 10.1186/s12889-019-7382-0 (PMC6683475; doi:10.1186/s12889-019-7382-0)
Supplement: Supplementary file 1 — Questionnaire. All questions asked in the questionnaire, in English. (DOCX 15 kb) [file 12889_2019_7382_MOESM1_ESM.docx]

**Additional file**

**File name: Additional file 1**

**Title: Questionnaire**

**Description: all questions asked in the questionnaire, in English**

Explanation of sun safety behaviors and sun exposure situations

*‘To protect the skin sufficiently against sunburn several protective measures can be taken. These sun safety practices can be divided in using sunscreen, wearing UV-protective clothing and seeking shade.’*

*Sufficient sunscreen use means: applying sunscreen on the skin*

- *For adults this means a SPF of at least 15, for children a SPF of at least 30*
- *Applying sunscreen 30 minutes prior to sun exposure*
- *Reapplicating sunscreen every two hours and more frequently while swimming or sweating*

*Sufficiently wearing protective clothing means: covering the skin with (among other things) clothing*

- *Wearing a sleeved t-shirt that cover the shoulders*
- *Wearing a hat or cap*
- *Wearing sunglasses (with UV-protective glasses)*

*Sufficiently shade seeking means:*

- *Seeking shade between 12 and 3 PM, while UVR is the strongest*

*As a parent, you can also support your child in performing sun safety behaviors him/herself. Supportive behavior consist of the following:*

- *Advising: giving your child advice on how to apply sun safety behaviors*
- *Facilitating: making sure your child is able to perform sun safety measures (e.g. buying sunscreen or sunglasses for your child to use)*
- *Checking: Actively checking whether your child applied sun safety behaviors correctly*

*‘Other than this, a distinction in sun exposure situations is made during the following questions in which you and/or your child are spending time outside.’*

- *A situation in which you or your child intentionally exposes to the sun. This is for example when you or your child goes to the swimming pool or the beach on a sunny day*
- *A situation in which you or your child incidentally exposes to the sun. This is for example when you or your child spends time outdoors playing, doing sports, bicycling or hiking*

Parental sun safety behavior

*‘The following questions are related to sun safety behaviors you perform during various activities.’*

*‘When your child went to the swimming pool or beach during the previous summer months … ’*

*‘To what extent did you make sure your child was sufficiently protected with sunscreen?’*

(Never – Always)

*‘To what extent did you make sure your child wore sufficient protective clothing?’*

(Never – Always)

*‘To what extent did you make sure your child was in shaded areas between 12 and 3 PM?’*

(Never – Always)

*‘When your child spent time outdoors during a sunny day while playing, doing sports, bicycle or hiking during the previous summer months … ’*

*‘To what extent did you make sure your child was sufficiently protected with sunscreen?’*

(Never – Always)

*‘To what extent did you make sure your child wore sufficient protective clothing?’*

(Never – Always)

*‘To what extent did you make sure your child was in shaded areas between 12 and 3 PM?’*

(Never – Always)

*‘The following questions are related to supporting your child while performing sun safety behaviors.’*

*‘When your child went to the swimming pool or beach during the previous summer months … ’*

*‘To what extent did you support your child so that he/she could protect him/herself sufficiently against sunburn?’*

(Never – Always)

*‘When your child spent time outdoors during a sunny day while playing, doing sports, bicycle or hiking during the previous summer months …’*

*‘To what extent did you support your child so that he/she could protect him/herself sufficiently against sunburn?’*

(Never – Always)

Children’s own sun safety behavior

*‘Depending on the age of a child, there are possible sun safety behaviors a child performs him or herself, with or without the presence of a parent or caretaker. We would like to know more about the extent to which your child is able to protect him or herself against sunburn.’*

*‘How often does your child perform sun safety behaviors as mentioned below?’*

*‘My child applies sunscreen sufficiently when he/she goes to the swimming pool or beach’*

(Never – Always)

*‘My child applies sunscreen sufficiently when he/she spends time outdoors during a sunny day, while playing, doing sports, bicycling or hiking’*

(Never – Always)

*‘My child puts on UV-protective clothing when he/she goes to the swimming pool or beach’*

(Never – Always)

*‘My child puts on UV-protective clothing when he/she spends time outdoors during a sunny day, while playing, doing sports, bicycling or hiking’*

(Never – Always)

*‘My child seeks shade when he/she goes to the swimming pool or beach’*

(Never – Always)

*‘My child seeks shade when he/she spends time outdoors during a sunny day, while playing, doing sports, bicycling or hiking’*

(Never – Always)
